# Histologic changes in immune-tolerant patients with chronic hepatitis B: a systematic review and meta-analysis

**DOI:** 10.1038/s41598-023-27545-z

**Published:** 2023-01-10

**Authors:** Zhiguo Li, Dongliang Yang, Yue Ge, Shu Song, Qin Lv, Yong’an Ye

**Affiliations:** 1 Department of Gastroenterology, Beijing Fengtai Hospital of Integrated Traditional and Western Medicine, Beijing, 100072 China; 2 Department of Math, Cangzhou Medical College, Cangzhou, Hebei China; 3 Institute of Liver Diseases, Beijing University of Chinese Medicine, Beijing, China

**Keywords:** Diagnosis, Pathology

## Abstract

The serological diagnostic criteria for the immune-tolerant (IT) phase have not been strictly defined and it is hard to determine an accurate rate for significant histologic changes among IT patients. The aim of this study was to establish a baseline rate of significant histologic changes and to determine the main characteristics of IT patients. We systematically searched PubMed, Embase, and Web of Science. Studies reporting liver biopsy results (inflammation grade or fibrosis stage) for adults with chronic hepatitis B virus (HBV) infection in the IT phase diagnosed by serological criterion were included to pool the rate of significant histologic changes. Studies that enrolled subjects with confirmed chronic HBV infection in the IT phase diagnosed by serological and liver biopsy criteria (dual criteria) were included to pool the mean values of main characteristics among IT patients. Of 319 studies screened, 15 were eventually included in the meta-analysis. The pooled rates of significant liver fibrosis and inflammatory activity for 10 studies were 10% (95% confidence interval [CI] 0.06–0.18) and 16% (95% CI 0.07–0.31), respectively. The pooled mean values of age, alanine aminotransferase level, HBV DNA level, and HBsAg level for another 5 studies with IT patients diagnosed by dual criteria were 30.7 years (95% CI 27.31–34.09), 26.64 IU/mL (95% CI 24.45–28.83), 8.41 log_10_ cp/mL (95% CI 7.59–9.23), and 4.24 log_10_ IU/mL (95% CI 3.67–4.82), respectively. Significant histologic changes were not rare events among IT patients. Strictly defined serological diagnostic criteria for the IT phase are warranted.

## Introduction

Hepatitis B virus (HBV) infection remains a serious global public health burden that affects approximately 240 million individuals worldwide and is the leading cause of cirrhosis and hepatocellular carcinoma (HCC)—the third primary cause of cancer deaths globally^1,2^. Chronic HBV infection typically undergoes dynamic phases depending on the host’s immune response and HBV replication^3^. The immune-tolerant (IT) phase, which is the earliest phase of chronic HBV infection, is characterized by high serum HBV DNA level, persistently normal alanine aminotransferase (ALT) levels, and no or minimal liver disease^4–6^.

High HBV DNA level is a major driving force that leads to hepatitis, thus increasing the risk of cirrhosis and HCC in IT patients^7,8^. Some studies reported HBV DNA integration, clonal hepatocyte expansion, and even significant histologic changes during the so-called IT phase in young patients with chronic HBV infection, as defined by their serological profile^9–11^. Furthermore, an initial research from Korea showed that the risk of HCC and death/transplantation was higher in untreated IT patients (defined by serological profile) than in treated immune-active patients^12^. Thus, considerable controversy has surrounded the treatment for IT patients for a few years^9,13–16^.

Antiviral therapy for patients during the IT phase has not been recommended by most practice guidelines because disease progression including histologic necroinflammation or fibrosis is not active at this stage^3,5,17^. Throughout the natural history of chronic HBV infection, serum HBV DNA titer is the highest during the IT phase, and there exists an undercurrent surge of immune clearance and inflammatory activity within the normal ALT range^18^. Previous studies showed that the rate of significant fibrosis in the IT phase can range from 0 to 46.8%, which is affected by various patient characteristics such as age, serum HBV DNA level, and HBsAg level^19,20^. In addition, major international guidelines have not yet reached a consensus on these characteristics^3,5,17^.

Because significant histologic changes are affected by virus–host interaction and immune response, accurately determining the rate of significant histologic changes in IT patients is difficult. The establishment of a baseline rate of significant histologic changes in IT patients would be important and crucial for clinical decision and patients’ benefit. Hence, we performed a systematic review and meta-analysis to address this issue. Additionally, we calculated the pooled mean values of main clinical parameters among IT patients diagnosed by dual criteria to determine the characteristics of IT patients.

## Methods

### Search strategy and selection criteria

This study is registered with PROSPERO (CRD42019125197). We systematically searched PubMed, Embase, and Web of Science from their inception to 1 October 2022 using a combination of search terms related to “chronic hepatitis B,” “immune tolerance,” and “liver biopsy.” Attempts were made to contact the corresponding authors for additional data. The references of all included studies were manually searched for additional eligible articles. The detailed search strategy is presented in Supplementary Methods.

Cross-sectional studies, observational cohort studies, and randomized controlled trials that reported liver biopsy results (inflammation grade or fibrosis stage) for adults with chronic HBV infection in the IT phase diagnosed by serological criterion were included to pool the rate of significant histologic changes. Studies that enrolled subjects with confirmed chronic HBV infection in the IT phase diagnosed by serological and liver biopsy criteria (dual criteria) were included to pool the mean values of main characteristics among IT patient.

The serological criterion for the IT phase was defined as high serum HBV DNA level (typically > 4.0 log_10_ IU/mL) and persistently normal (upper limit of normal [ULN] approximately 40 IU/mL). The liver biopsy criterion for the IT phase was defined as no or minimal liver disease (liver inflammation or fibrosis).

The following studies were excluded from the analysis: studies with a sample size of < 10 individuals; studies that included subjects with other overlapping causes of hepatitis and fibrosis, such as hepatitis C and hepatitis D; studies that included subjects co-infected with human immunodeficiency virus; studies that included subjects who had prior HCC, underwent liver transplantation, or received chemotherapy; pediatric studies, although we included studies enrolling subjects with age as low as 9 years; studies with insufficient data for extraction; case–control studies; and letters, reviews, posters, editorials, dissertations, and conference abstracts.

### The diagnostic criteria for liver biopsy

The histological evaluation of the included studies consisted of Knodell system, Ishak system, Metavir system, Scheurer system, Batts-ludwig system and Chinese system. The diagnostic criteria for liver biopsy are based on different histological evaluation systems: (1) Knodell system^21^, where the hepatic activity index (HAI) was used to describe the hepatocellular necroinflammation activity with scores range from 0 to 22. HAI score ≥ 9 indicated significant inflammation, and HAI score 0–8 indicated no or minimal liver inflammation. (2) Ishak system^22^ of fibrosis, where the severity of fibrosis was graded from stage 0 to stage 6. Stage 0–2 indicated no or minimal liver fibrosis, and stage 3–6 indicated significant liver fibrosis. (3) Scheuer system^23^, where the severity of necroinflammation was graded from G0 to G4 and stage of fibrosis was graded from S0 to S4. S0–S1 indicated no or minimal liver fibrosis, and S2–S4 indicated significant liver fibrosis. G0–G1 indicated no or minimal liver inflammation, and G2–G4 indicated significant liver inflammation. (4) METAVIR system^24^, where the severity of necroinflammation was graded from A0 to A3 and stage of fibrosis was graded from F0 to F4. A0–A1 indicated no or minimal liver inflammation, and A2-A4 indicated significant liver inflammation. F0–F1 indicated no or minimal liver fibrosis, and F2–F4 indicated significant liver fibrosis. (5) Batts-Ludwig system^25^ and Chinese system^26^, which include five categories (0 to 4) separately for both inflammation grade and fibrosis stage. Grade 0–1 indicated no or minimal liver inflammation, and grade 2–4 indicated significant liver inflammation. Stage 0–1 indicated no or minimal liver fibrosis, and stage 2–4 indicated significant liver fibrosis.

### Selection process and data extraction

Citations were merged in EndNote version X8 (Thomson Reuters, New York, NY, USA) to facilitate management. Two reviewers independently examined the titles and abstracts in duplicate to identify potentially eligible studies and subsequently reviewed the full texts to identify studies in accordance with the inclusion and exclusion criteria. We extracted data from the selected studies using a standardized form. Two data summary tables were prepared and compared for concordance. Disagreements were resolved by a third reviewer. The following data were extracted from all eligible studies: first author, publication year, study location, study design, sample size, ULN for ALT, criteria for immune tolerance, sex, ALT level, age, HBV DNA titer, HBsAg level, standard for liver biopsy, and liver biopsy results.

### Outcomes

The predetermined primary outcome was the rate of significant histologic changes among IT subjects. Significant histologic changes were defined as significant liver fibrosis or significant inflammatory activity on liver biopsy. The secondary outcome was the mean values of main characteristics among IT patients diagnosed by dual criteria.

### Quality assessment

The Newcastle–Ottawa quality assessment scale was used to evaluate the quality of included studies^27^. Studies were scored according to the following three items: patient selection (four stars), comparability of study groups (two stars), and assessment of outcome/exposure (three stars). With this scale (a maximum of nine stars), a score rating system was employed to indicate the quality of each study. Scores of 0–3, 4–6, and ≥ 7 signified poor, fair, and high quality, respectively.

### Statistical analysis

We calculated the pooled rate of significant histologic changes among subjects in the IT phase using random-effects meta-analysis. In addition, we pooled the mean values of key clinical parameters (ALT level, age, HBV DNA level, and quantitative HBsAg level) among IT patients diagnosed by dual criteria. Cochran’s Q statistic (with heterogeneity < 0.10 suggesting statistical significance) and *I*^2^ statistic (with *I*^2^ > 75.0% representing substantial heterogeneity, 50.0% ≤ *I*^2^ ≤ 75.0% representing moderate heterogeneity, and *I*^2^ < 50% representing low heterogeneity) were adopted to qualitatively and quantitatively evaluate heterogeneity across studies, respectively.

Sources of heterogeneity were mainly investigated using subgroup analyses by stratifying preplanned variables, including study location, study type, mean age, and ALT level. Furthermore, a sensitivity analysis was performed by excluding studies that enrolled subjects aged < 18 years, studies with sample sizes of < 50 individuals, and studies that did not definitely define HBV DNA titer in IT patients. Publication bias was assessed using a funnel plot and Egger’s test^28^.

Statistical significance level was set at two-sided *P* < 0.05, unless otherwise specified. All statistical analyses were performed using the “meta” package of R software version 3.5.2 (R Software Foundation, Vienna, Austria). We used metaprop to pool the rate of significant histologic changes among IT patients and metamean to pool the mean values of main characteristics among IT patients diagnosed by dual criteria.

## Results

**Figure 1:**
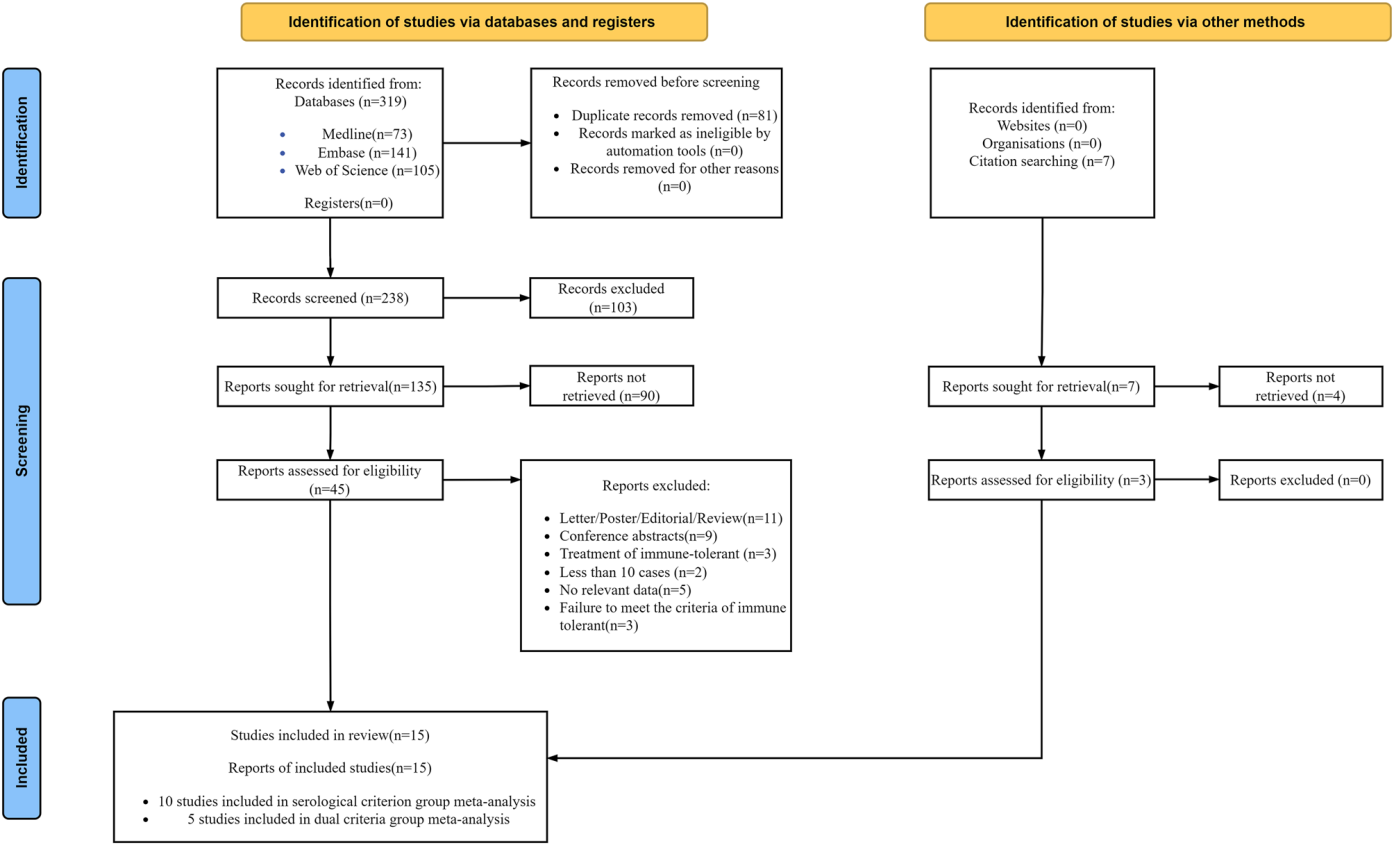
Flow diagram of literature search and study selection.

The selection process is shown in Fig. 1. The initial search yielded 319 records from the three databases. A total of 45 citations were thought to be potentially relevant after reviewing the titles and abstracts. Nevertheless, 33 citations were excluded after carefully reading the full texts. Three studies were considered eligible for inclusion in the hand-searching process. Finally, 15 studies fulfilled the inclusion criteria and were included in the meta-analysis^19,29–42^. These studies cumulatively reported 1,588 subjects.

### Study characteristics and quality assessment

The characteristics of the included studies are summarized in Table 1. Among the included studies, 10 studies^19,29–36,42^ reported the rate of significant liver disease among IT patients according to the serological criterion, whereas the other 5 studies^37–41^ reported the mean values of main characteristics among IT patients diagnosed by dual criteria. The included studies were from 5 countries, with 13 studies conducted in Asia and the remaining studies conducted in France^19^ and the United States^31^. All of the included studies defined ALT level within the ULN in IT patients. The sample size of the included studies ranged from 10 to 455 subjects, with the proportion of males ranging from 21.6 to 83.3%. One study included subjects younger than 18 years of age^29^. The mean or median age and ALT level ranged from 18 to 39 years and from 21 to 33 IU/mL, respectively.

**Table 1:** Characteristics of the included studies and patients.

First author	Year	Country	Cohort size	ALT (ULN)	Serological criteria for immune tolerance			Male	Mean age,y	ALT (IU/mL)	HBV DNA (log_10_IU/mL)	HBsAg (log_10_IU/mL)
					HBV DNA	Normal ALT duration	HBeAg					
Andreani T^19^	2007	France	40	35	> 10^7^ cp/ml	Normal ALT level within a half year	+	21	29.2^a^	21^a^	8.93^a^*	–
Li J^29^	2007	China	54	40	> 10^5^ cp/ml	Normal ALT level Within one year	+	31	24.93	–	7.52^a^*	–
Park JY^30^	2008	Korea	27	40	High level	Normal ALT level at least one year	+	19	28.8	22	9.18^a^*	–
Wang CC^31^	2008	USA	22	42/31^b^	> 10^5^ cp/ml	Normal ALT level at least 2 times	+	10	28^a^	28^a^	8.26^a^*	–
Wan RJ^32^	2015	China	125	40	High level	Normal ALT level	+	82	33.79	26.94	6.59*	–
Wu JZ^33^	2017	China	102	40	High level	Normal ALT level	+	40	27.42	27.48	7.58	–
Liu HY^34^	2018	China	22	40	High level	Normal ALT level	+	14	30^a^	30^a^	7.74*	–
Xing YF^35^	2018	China	455	40	10^5^–10^9^ IU/ml	Normal ALT level within one year	+	287	34.86	27.29	8.14^a^	–
Zhang P^36^	2018	China	288	40	2 × 10^4^–10^8^ IU/ml	Normal ALT level within one year	+	171	37.6	30.11	–	–
Hui CK^37^	2007	China	57	53/31^b^	> 10^7^ cp/mL	Normal ALT level within 18 months	+	34	31^a^	30^a^	9.81*	–
Li WJ^38^	2014	China	10	–	> 10^7^ IU/mL	Normal ALT level	+	7	18^a^	33^a^	7.95	4.35^a^
Chen EQ^39^	2017	China	59	50/38^b^	High level	Normal ALT level	+	40	31.58	24.93	7.70	4.48
Singh AK^40^	2018	India	30	–	High level	Normal ALT level	+	25	39	26	7.01	3.59^a^
Wang J^41^	2018	China	24	–	High level	Normal ALT level	+	14	30^a^	24^a^	6.78*	4.71
Hu AR^42^	2021	China	273	40	> 2 × 10^7^ IU/mL	Normal ALT level within a year	+	150	33.1	22.98	8	–

^a^Median; ^b^High value for male and low value for female; *log_10_copy/mL; USA, the United States of America.

Ten studies reported events and rate of significant histologic changes on liver biopsy (Table 2). The staging and grading systems involved in these studies were the METAVIR system, Scheuer system, Batts–Ludwig system, Ishak fibrosis score, Knodell histology activity index, and Chinese system. The rates of significant liver fibrosis and inflammatory activity ranged from 0 to 31.8% and 59.8%, respectively. The overall rate of significant histologic changes was absent because most studies did not report the overall events. Quality assessment was performed on the 15 included studies, and no study showed high risk of bias (details shown in Supplementary Table 1).

**Table 2:** Events and rate of significant histological changes.

First author	Cohort size	Staging system	Significant liver fibrosis (n, %)	Grading system	Significant inflammation activity (n, %)
Andreani T^19^	40	Metavir system	0	Metavir system	2 (5%)
Li J^29^	54	Scheuer system	6 (11.1%)	Scheuer system	24 (44.4%)
Park JY^30^	27	Metavir system	5 (18.5%)	Metavir system	1 (3.7%)
Wang CC^31^	22	Batts-Ludwig system	7 (31.8%)	Batts-Ludwig system	6 (27.3%)
Wan RJ^32^	125	Scheuer system	38 (30.4%)	Scheuer system	46 (36.8%)
Wu JZ^33^	102	Scheuer system	2 (2.0%)	Scheuer system	61 (59.8%)
Liu HY^34^	22	Scheuer system	2 (9.1%)	Scheuer system	0
Xing YF^35^	455	Ishak's fibrosis score	46 (10.1%)	Knodell HAI system	25 (5.5%)
Zhang P^36^	288	Scheuer system	27 (9.4%)	Scheuer system	50 (17.4%)
Hu AR^42^	273	Chinese system	30 (11%)	Chinese system	43 (15.8%)

### Pooled rate of significant histologic changes

**Figure 2:**
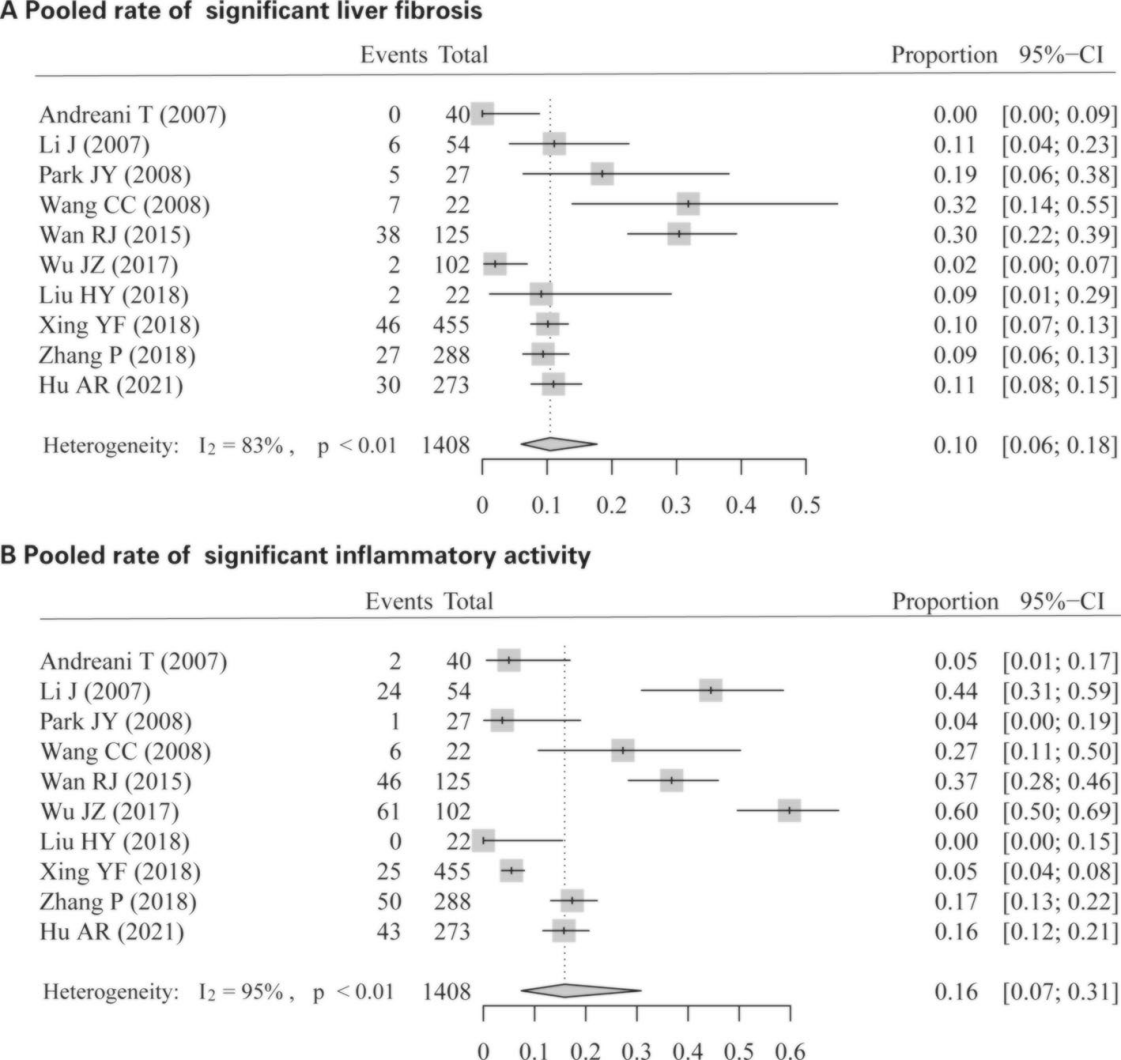
Pooled rate of significant histologic changes (significant liver fibrosis and inflammatory activity).

Of 1408 patients in 10 studies, 163 (11.6%) and 258 (18.3%) showed significant liver fibrosis and inflammatory activity. The pooled rates of significant liver fibrosis and inflammatory activity in these 10 studies were 10% (95% confidence interval [CI] 0.06–0.18, *I*^2^ = 83%; Fig. 2A) and 16% (95% CI 0.07–0.31, *I*^2^ = 95%; Fig. 2B), respectively.

### Pooled mean values of main characteristics among IT patients diagnosed by dual criteria

**Figure 3:**
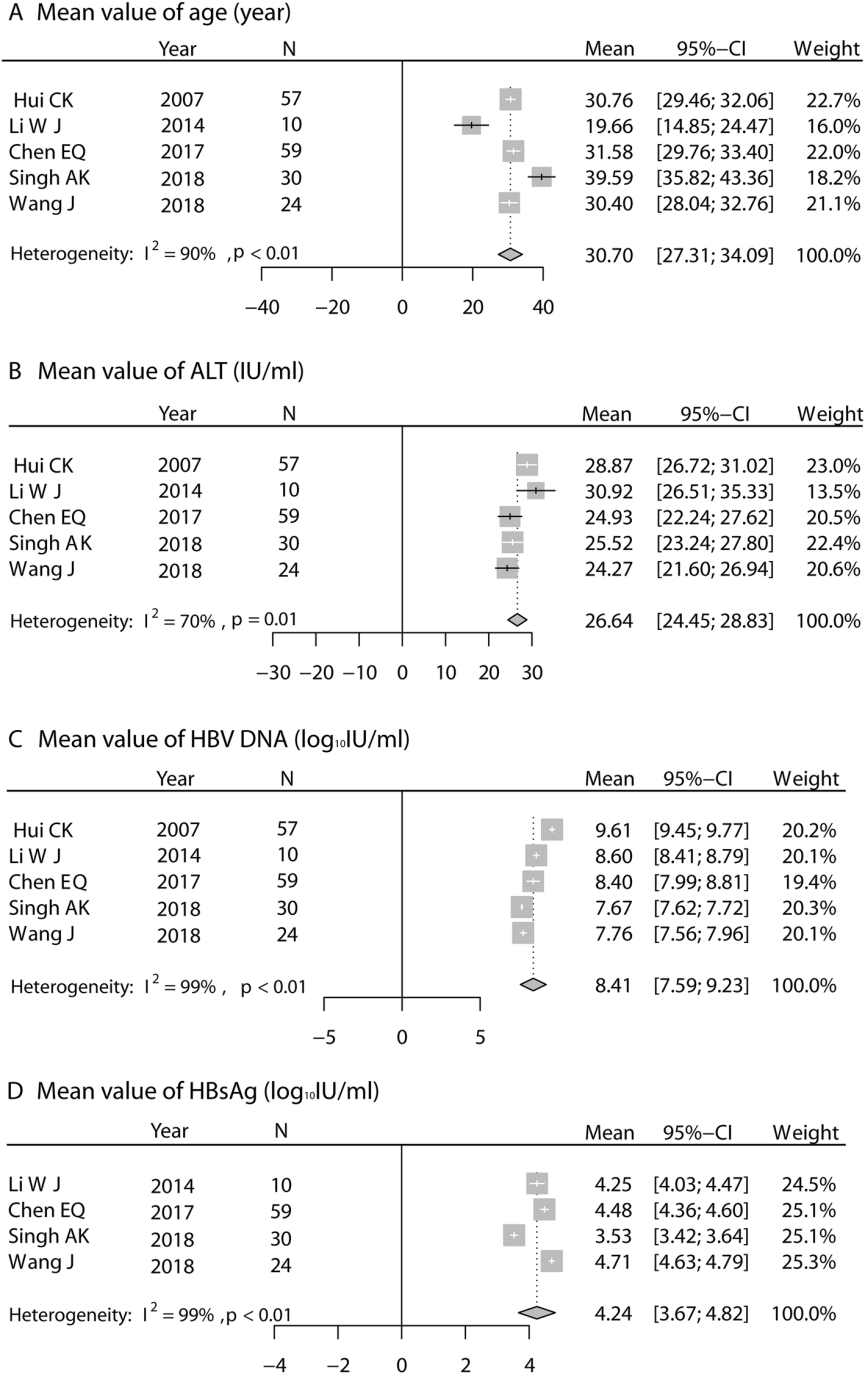
Pooled mean values of main characteristics among IT patients diagnosed by dual criteria.

In 180 patients from 5 studies, the pooled mean values of age, ALT level, HBV DNA level, and HBsAg level among IT patients diagnosed by dual criteria were 30.7 years (95% CI 27.31–34.09, *I*^2^ = 90%), 26.64 IU/mL (95% CI 24.45–28.83, *I*^2^ = 70%), 8.41 log_10_ cp/mL (95% CI 7.59–9.23, *I*^2^ = 99%), and 4.24 log_10_ IU/mL (95% CI 3.67–4.82, *I*^2^ = 99%), respectively (Fig. 3).

### Rate of significant histologic changes according to baseline demographic and clinical characteristics

The pooled rate of significant liver fibrosis for Asia and Western countries was 11% (95% CI 0.07–0.17) and 3% (95% CI 0.00–0.85), respectively, albeit without statistically significant difference (Table 3). The mean age in 9 studies^19,29–32,34–36,42^ was 32 years, and the pooled rate of significant liver fibrosis was similar between studies that included subjects with a mean age of < 32 years (11%; 95% CI 0.04–0.25) and studies that included subjects with a mean age of > 32 years (13%; 95% 0.08–0.22), albeit without statistically significant difference. The mean ALT level in 8 studies^19,30–32,34–36,42^ was 26 IU/mL, and the pooled rate of significant liver fibrosis for studies with ALT level < 26 IU/mL (8%; 95% CI 0.02–0.25) was slightly lower than that for studies with ALT level > 26 IU/mL (16%; 95% CI 0.09–0.26), albeit without statistically significant difference.

**Table 3:** Rate of significant histologic changes according to baseline demographic and clinical characteristics.

Subgroups	Number of studies	Significant liver fibrosis rate (95% CI)	Heterogeneity I^2^ (%)	P*	Significant inflammation activity rate (95% CI)	Heterogeneity I^2^ (%)	P*
**Location**							
Asia	8^29,30,32–36,42^	11% (0.07–0.17)	85	0.62	17% (0.07–0.35)	96	0.70
Western	2^19,31^	3% (0.00–0.85)	0		13% (0.03–0.37)	80	
**Mean age**							
< 32	5^19,29–31,34^	11% (0.04–0.25)	27	0.65	9% (0.02–0.33)	79	0.48
> 32	4^32,35,36,42^	13% (0.08–0.22)	92		16% (0.08–0.30)	96	
**Mean ALT**							
< 26	3^19,30,42^	8% (0.02–0.25)	0	0.31	9% (0.03–0.22)	61	0.54
> 26	5^31,32,34–36^	16% (0.09–0.26)	90		13% (0.05–0.30)	94	

*Heterogeneity between group.

The pooled rate of significant inflammatory activity was slightly higher for Asia (17%; 95% CI 0.07–0.35) than for Western countries (13%; 95% CI 0.03–0.37), albeit without statistically significant difference. The pooled rate of significant inflammatory activity for studies that included subjects with a mean age of < 32 years (9%; 95% CI 0.02–0.33) was a little lower than that for studies with a mean age of > 32 years (16%; 95% CI 0.08–0.30), albeit without statistically significant difference. The pooled rate of significant inflammatory activity for studies with ALT level < 26 IU/mL (9%; 95% CI 0.03–0.22) was lower than that for studies with ALT level > 26 IU/mL (13%; 95% CI 0.05–0.30), albeit without statistically significant difference.

Analysis of study type was not performed because of a lack of primary data. There were no publication biases in the meta-analysis of the rates of significant liver fibrosis and inflammatory activity according to the funnel plot (Supplementary Figs. 1 and 2) using Egger’s test (*P* = 0.66 and 0.71, respectively).

### Sensitivity analysis

We performed 3 sensitivity tests for 10 studies (Supplementary Figs. 3–5). In the first sensitivity test in which studies that enrolled subjects aged < 18 years were excluded, the pooled rate of significant liver fibrosis remained the same (10%; 95% CI 0.05–0.19, *I*^2^ = 85%), whereas the pooled rate of significant inflammatory activity slightly lowered (14%; 95% CI 0.06–0.28, *I*^2^ = 95%). Nonetheless, heterogeneity remained high. In the second sensitivity test, we excluded studies that did not definitely define HBV DNA titer in IT patients. The pooled rate of significant liver fibrosis remained the same at 10% (95% CI 0.09–0.12, *I*^2^ = 48%) with moderate heterogeneity. The pooled rate of significant inflammatory activity remained similar at 15% (95% CI 0.08–0.28, *I*^2^ = 92%) with high heterogeneity. Finally, we excluded studies with sample sizes of < 50 individuals. For the remaining studies, the pooled rate of significant liver fibrosis remained the same (10%; 95% CI 0.06–0.18, *I*^2^ = 89%), whereas the pooled rate of significant inflammatory activity became higher (25%; 95% CI 0.12–0.44, *I*^2^ = 97%), respectively. Nonetheless, heterogeneity remained high.

## Discussion

To our knowledge, this study is the first systematic effort to evaluate the rate of significant histologic changes among IT patients. In this systematic review and meta-analysis, we determined that the pooled rates of significant liver fibrosis and inflammatory activity were 10% and 16%, respectively. Significant histologic changes are not rare events among IT patients. Our finding is in agreement with that of a large-scale prospective multicenter study^35^, in which the rates of significant liver fibrosis and inflammatory activity were 9.4% and 17.4%, respectively. However, the substantial heterogeneity of our results requires that they should be interpreted with caution.

The pooled rates of significant liver fibrosis and inflammatory activity were higher in Asia than in Western countries, albeit without statistically significant difference. This could have resulted from the limited study size and quantity in Western countries and the high prevalence of HBV infection in Asia, which accounted for more than 57% of all HBsAg-positive infections^43^. The American practice guideline has recommended lower ALT levels to define the IT phase^5^. Along with the findings of others^30,34^, our analysis confirmed that a lower ALT level could predict a lower rate of significant histologic changes, albeit without statistically significant difference in our study.

It is well known that younger IT patients generally have high HBV DNA titer and show minimal hepatitis activity^15^. The subgroup analysis of age in our study revealed that younger patients were less likely to have significant liver fibrosis and significant inflammatory activity. This result was similar to the finding of the study^35^ mentioned above, which performed univariate analysis of factors and histologic disease. In this previous study, younger patients were less likely to develop significant liver fibrosis and inflammatory activity, though significant difference in significant inflammatory activity was not observed^35^.

By combining the data of IT patients diagnosed by dual criteria, we determined the main characteristics of IT patients. The pooled mean values of age, ALT level, HBV DNA level, and HBsAg level were 30.7 years, 26.64 IU/mL, 8.41 log_10_ cp/mL, and 4.24 log_10_ IU/mL, respectively, which were consistent with the definition of IT in most practice guidelines^3,5,17^. Although our study indicated a mean age of 30.7 years, IT loss often occurs at a mean age of 30–35 years (40 years in 90% of individuals)^4^. In a French study that defined HBV DNA titer of more than 10^7^ cp/mL among IT patients, liver biopsy showed extremely low histologic disease rate^19^. The pooled mean HBV DNA value in our study further shows that we need to strictly define HBV DNA titer in the IT phase to avoid the unnecessary performance of liver biopsy^44^. Similar to other studies, our study indicated that a high HBsAg level (typically > 4.0 log_10_ IU/mL) is also an important feature of the IT phase^45–47^.

Our study has some limitations that warrant attention. First, serological diagnostic criteria for the IT phase in the included studies, especially HBV DNA, were not uniform, which would affect the rate of significant histologic changes. Major international guidelines have not reached a consensus on serological diagnostic criteria for the IT phase^3,5,17^. For instance, the American practice guideline has recommended a very high HBV DNA level (typically > 10^6^ IU/mL) to define the IT phase, whereas the recent European and Asian–Pacific practice guideline has recommended an HBV DNA level > 10^7^ IU/mL; furthermore, the Chinese practice guideline recommended a very high HBV DNA level (typically > 10^5^ IU/mL)^3,17,48^. In our study, most of the included studies defined a high HBV DNA level for IT patients, but we performed sensitivity analysis of only studies with definitely define HBV DNA titer in IT patients and found a similar pooled estimate as the main result.

Second, there was insufficient diversity in represented countries, particularly in Asia. Studies from China accounted for more than half of all included studies, and most studies were from Asia. According to a recent study, Asian countries have a high prevalence of HBV infection. Moreover, limited cases were included in our study. This could be because liver biopsy is an invasive examination that is difficult for subjects to accept. Noninvasive methods for the assessment of liver fibrosis such as liver stiffness measurements prove to be excellent with respect to performance and are widely available^49,50^. However, data on noninvasive methods for IT patients are still not available. Finally, the pooled mean values of main characteristics and pooled rates of significant liver fibrosis and inflammatory activity should be interpreted with caution because of high heterogeneity, despite our attempt to reduce it using subgroup and sensitivity analysis.

## Conclusion

In this systematic review and meta-analysis, we determined that significant histologic changes were not rare events among IT patients. Strictly defined serological diagnostic criteria for the IT phase are warranted to avoid the occurrence of significant histologic changes during this period.

## Supplementary Information

Supplementary Legends.

Supplementary Figure 1.

Supplementary Figure 2.

Supplementary Figure 3.

Supplementary Figure 4.

Supplementary Figure 5.

Supplementary Table 1.

Supplementary Information 1.

Supplementary Information 2.

## Data Availability

The data that support the findings of this study are available in the paper and its Supplementary Information, or from the corresponding authors upon reasonable request.
