# Supplementary material for: Histologic changes in immune-tolerant patients with chronic hepatitis B: a systematic review and meta-analysis
Source: Sci Rep. 2023 Jan 10;13:469. doi: 10.1038/s41598-023-27545-z (PMC9831999; doi:10.1038/s41598-023-27545-z)
Supplement: Supplementary file 1 — Supplementary Legends. [file 41598_2023_27545_MOESM1_ESM.docx]

# Figure legend

**Supplementary Fig. 1.** Begg funnel plot of included studies reported rate of significant liver fibrosis.

**Supplementary Fig. 2.** Begg funnel plot of included studies reported rate of significant inflammatory activity.

**Supplementary Fig. 3.** Sensitivity analysis excluded studies with subjects less than18 years.

**Supplementary Fig. 4.** Sensitivity analysis excluded studies did not definitely define HBV DNA titer in IT patients.

**Supplementary Fig. 5.** Sensitivity analysis excluded studies with sample less than 50.
