# Supplementary figures and images for: Histologic changes in immune-tolerant patients with chronic hepatitis B: a systematic review and meta-analysis
Source: Sci Rep. 2023 Jan 10;13:469. doi: 10.1038/s41598-023-27545-z (PMC9831999; doi:10.1038/s41598-023-27545-z)

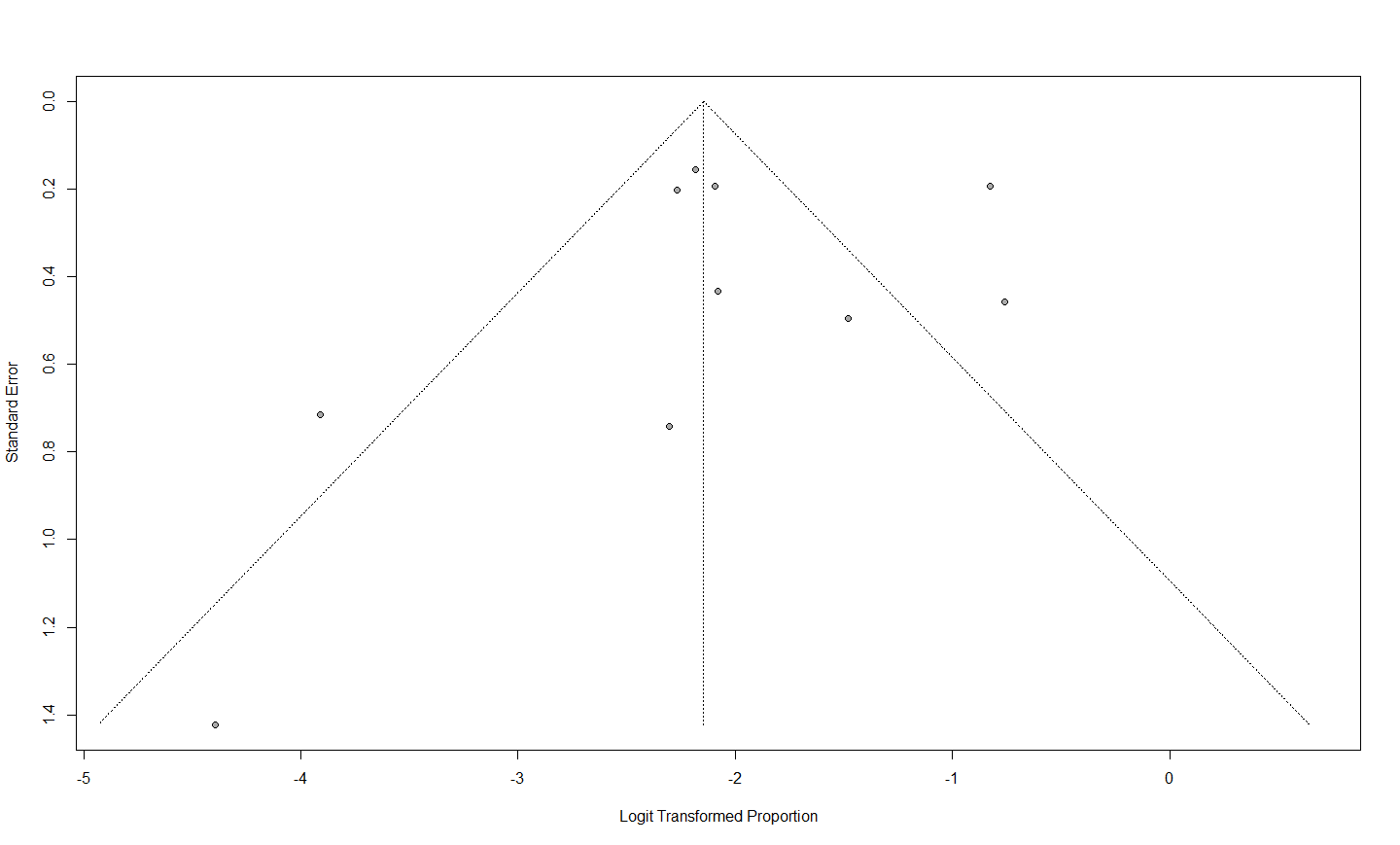

Supplement: Supplementary file 2 — Supplementary Figure 1. [file 41598_2023_27545_MOESM2_ESM.tiff]

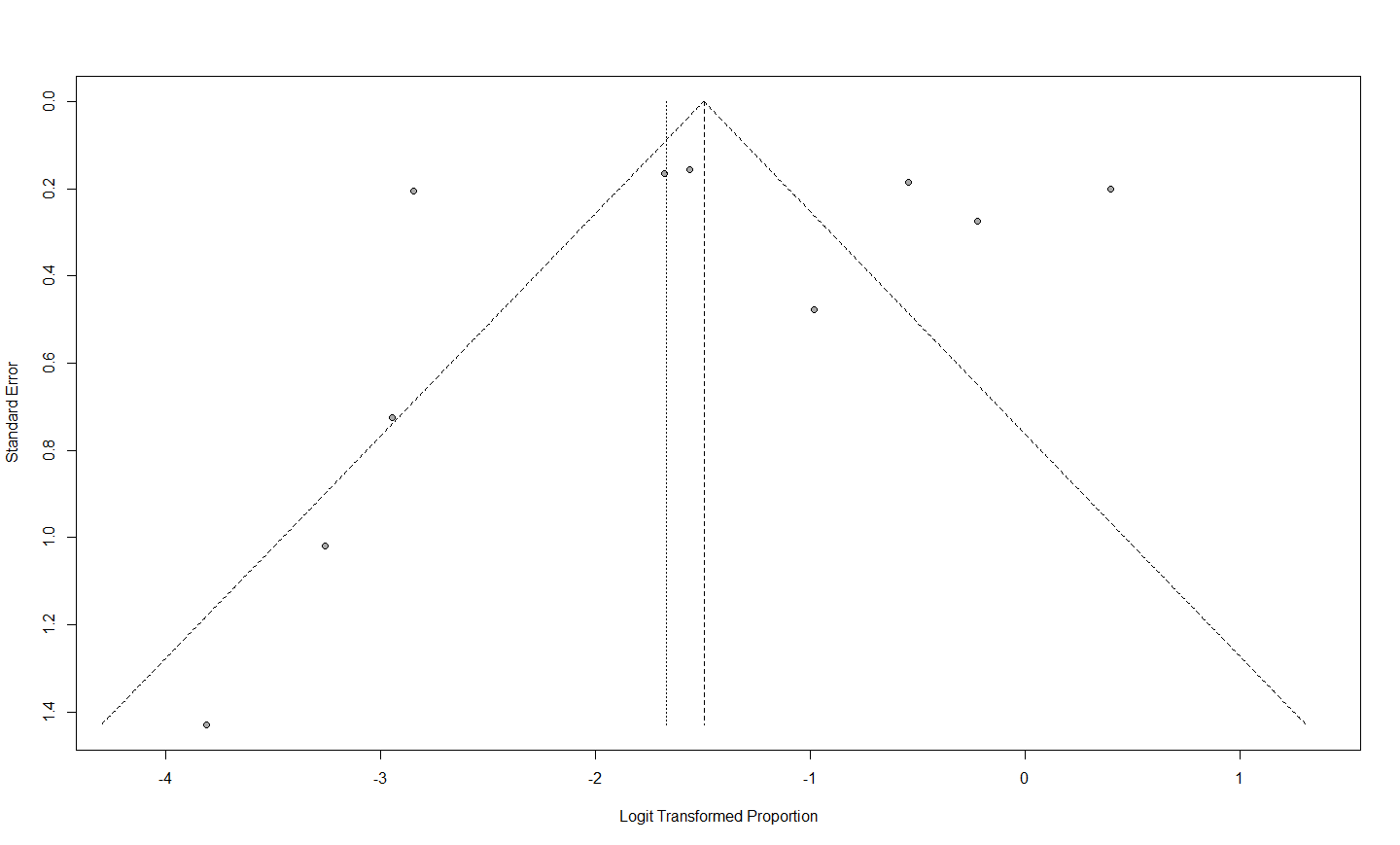

Supplement: Supplementary file 3 — Supplementary Figure 2. [file 41598_2023_27545_MOESM3_ESM.tiff]

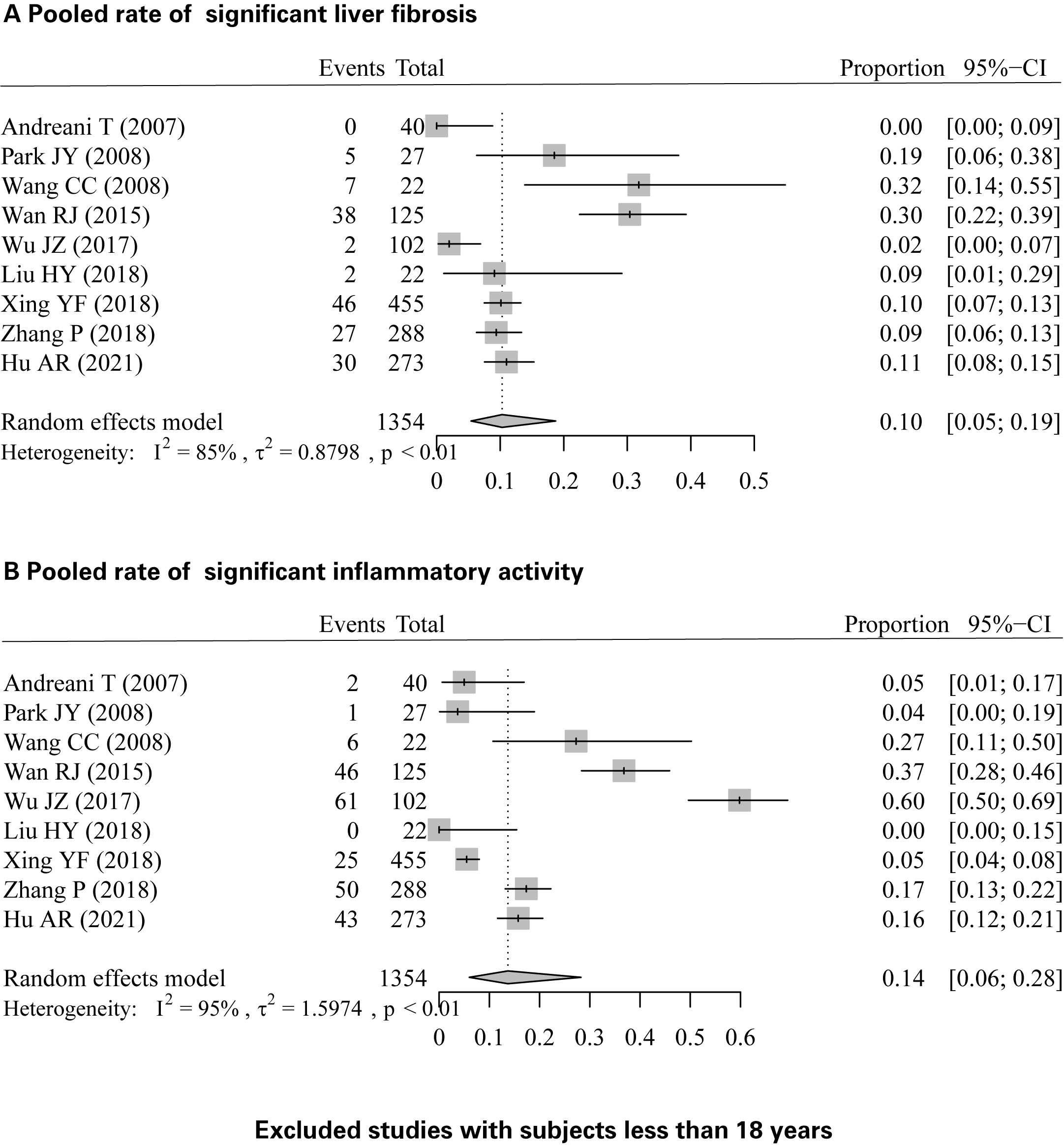

Supplement: Supplementary file 4 — Supplementary Figure 3. [file 41598_2023_27545_MOESM4_ESM.tif]

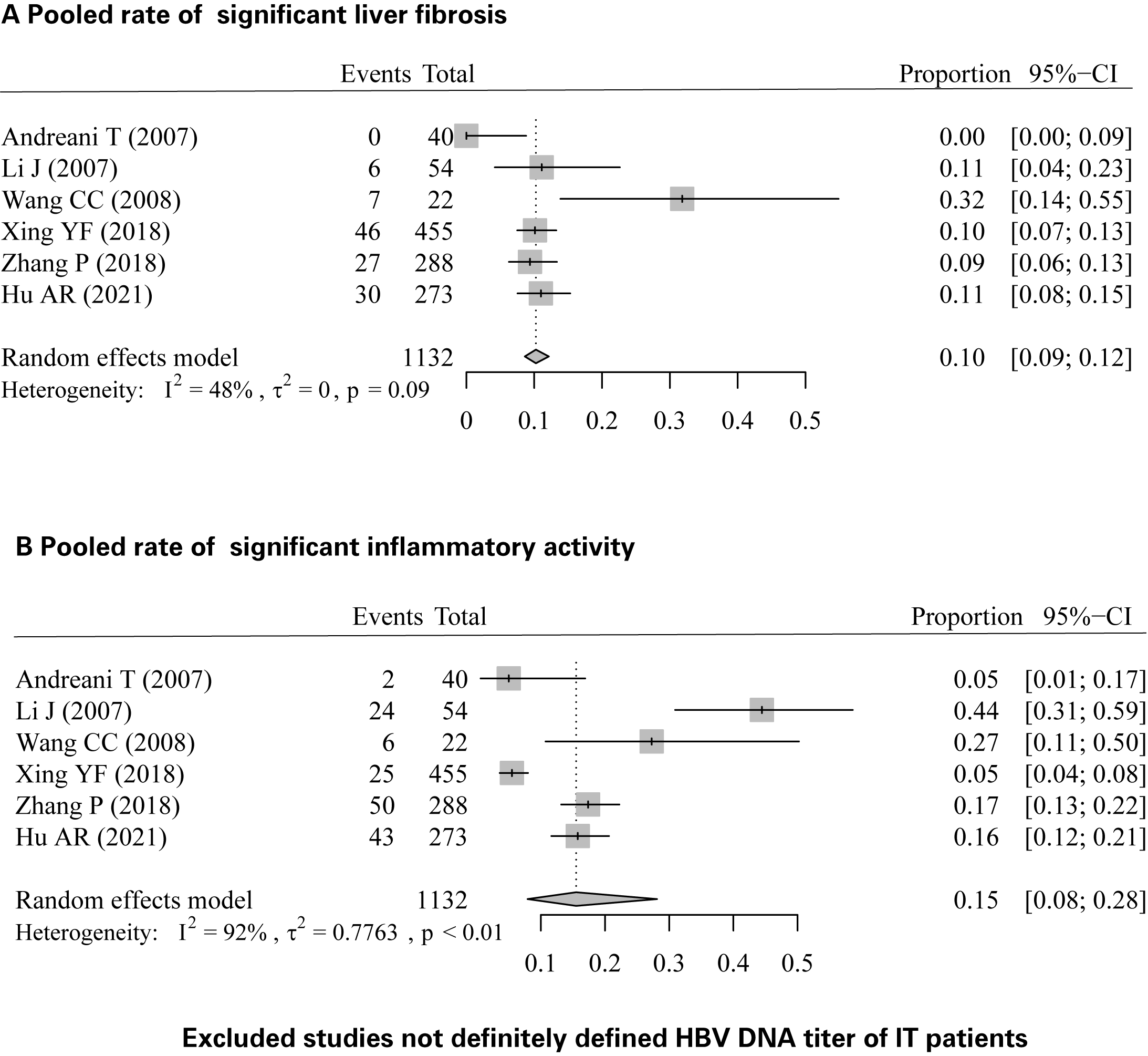

Supplement: Supplementary file 5 — Supplementary Figure 4. [file 41598_2023_27545_MOESM5_ESM.tif]

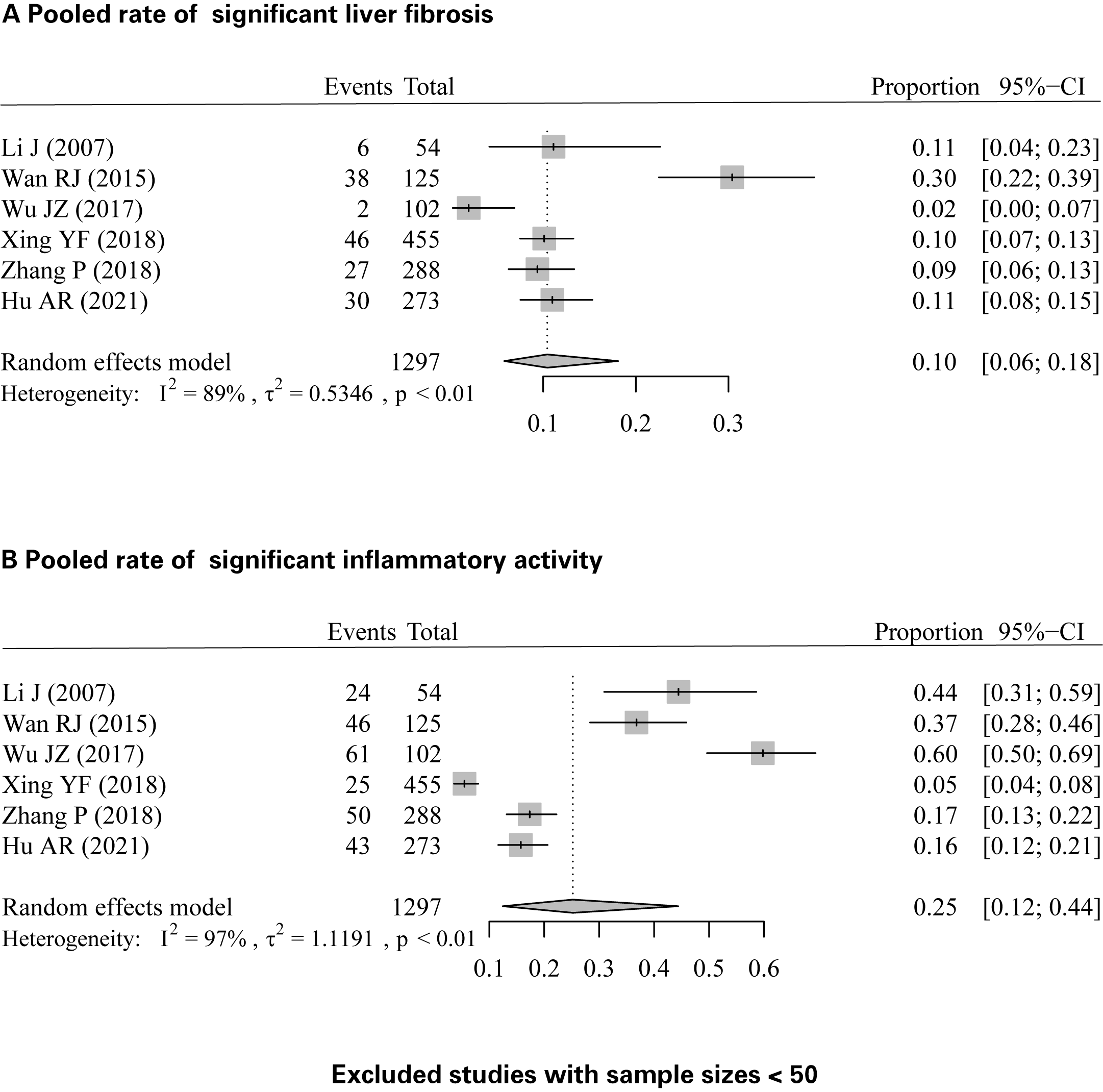

Supplement: Supplementary file 6 — Supplementary Figure 5. [file 41598_2023_27545_MOESM6_ESM.tif]
