# Supplementary material for: Histologic changes in immune-tolerant patients with chronic hepatitis B: a systematic review and meta-analysis
Source: Sci Rep. 2023 Jan 10;13:469. doi: 10.1038/s41598-023-27545-z (PMC9831999; doi:10.1038/s41598-023-27545-z)
Supplement: Supplementary file 7 — Supplementary Table 1. [file 41598_2023_27545_MOESM7_ESM.docx]

| **Supplementary Methods (**Detailed search strategy**)**  ***Pubmed*** | |
| --- | --- |
| **Number** | **Search terms** |
| #1 | Search chronic hepatitis B [MeSH Terms] |
| #2 | Search hepatitis B virus [Title/Abstract] |
| #3 | Search HBV[Title/Abstract] |
| #4 | #1 OR #2 OR #3 |
| #5 | Search immune tolerance [Title/Abstract] |
| #6 | Search immune-tolerance [Title/Abstract] |
| #7 | Search immune tolerant [Title/Abstract] |
| #8 | Search immune-tolerant [Title/Abstract] |
| #9 | #5 OR #6 OR #7 OR #8 |
| #10 | Search patholog*[Title/Abstract] |
| #11 | Search biopsy [Title/Abstract] |
| #12 | Search histolog*[Title/Abstract] |
| #13 | Search "liver biopsy"[Title/Abstract] |
| #14 | #10 OR #11 OR #12 OR #13 |
| #15 | #4 AND #9 AND #14 |

***EMBASE***

| **Number** | **Search terms** |
| --- | --- |
| #1 | 'chronic hepatitis b'/exp |
| #2 | 'hepatitis b'/exp |
| #3 | 'hepatitis b virus':ta,ab,kw |
| #4 | 'hepatitis b':ta,ab,kw |
| #5 | 'chronic hepatitis b':ta,ab,kw |
| #6 | #1 OR #2 OR #3 OR #4 OR #5 |
| #7 | 'immunological tolerance'/exp |
| #8 | 'immune tolerance':ta,ab,kw |
| #9 | 'immune-tolerance':ta,ab,kw |
| #10 | 'immune-tolerant':ta,ab,kw |
| #11 | 'immune tolerant':ta,ab,kw |
| #12 | 'immune tolerant' |
| #13 | 'immune tolerance' |
| #14 | 'liver histology'/exp |
| #15 | 'liver histology':ta,ab,kw |
| #16 | 'histology':ta,ab,kw |
| #17 | 'pathology'/exp |
| #18 | 'pathology':ta,ab,kw |
| #19 | 'patholog*':ta,ab,kw |
| #20 | 'histolog*':ta,ab,kw |
| #21 | #7 OR #8 OR #9 OR #10 OR #11 OR #12 OR #13 |
| #22 | #14 OR #15 OR #16 OR #17 OR #18 OR #19 OR #20 |
| #23 | #6 AND #21 AND #22 |

***Web of Science***

TS=(chronic hepatitis B OR HBV OR hepatitis B virus) AND TS=(immune tolerance OR immune tolerant) AND TS=（liver biopsy OR histolog* OR patholog*）

| **Supplementary Table 1** Newcastle-Ottawa Scale (NOS) Quality Assessment of Included Articles | | | | |
| --- | --- | --- | --- | --- |
| Study/Years of Publication | Selection Score (4 Points) | Comparability Score (2 Points) | Outcome Score (3 Points) | Total Score (9 Points) |
| Andreani T (2007) | 2 | 1 | 3 | 6 |
| Li J (2007) | 4 | 1 | 1 | 6 |
| Park JY (2008) | 4 | 1 | 1 | 6 |
| Wang CC (2008) | 3 | 1 | 1 | 5 |
| Wan RJ (2015) | 3 | 1 | 1 | 5 |
| Wu JZ (2017) | 3 | 1 | 1 | 5 |
| Liu HY (2018) | 4 | 1 | 1 | 6 |
| Xing YF (2018) | 4 | 2 | 1 | 6 |
| Zhang P (2018) | 4 | 2 | 1 | 6 |
| Hui CK (2007) | 4 | 2 | 3 | 9 |
| Li WJ (2014) | 4 | 2 | 1 | 7 |
| Chen EQ (2017) | 4 | 2 | 1 | 7 |
| Singh AK (2018) | 4 | 2 | 1 | 7 |
| Wang J (2018) | 4 | 2 | 1 | 7 |
| H AR (2021) | 4 | 2 | 1 | 7 |
